# Cervical mature teratoma 17 years after initial treatment of testicular teratocarcinoma: report of a late relapse

**DOI:** 10.1186/1477-7819-5-1

**Published:** 2007-01-04

**Authors:** Ramesh Omranipour, Mina Alavion

**Affiliations:** 1 Department Of Surgical Oncology, Cancer Institute, Tehran University Of Medical Science, Tehran, Iran

## Abstract

**Background:**

Late relapses of testicular germ cell tumor are uncommon. We report a case of cervical mature teratoma appeared 17 years after treatment of testicular teratocarcinoma.

**Case presentation:**

A 20- year- old patient underwent left sided orchiectomy followed by systemic therapy and retroperitoneal residual mass resection in 1989. He remained in complete remission for 200 months. In 2005 a huge left supraclavicular neck mass with extension to anterior mediastinum appeared. Radical surgical resection of the mass was performed and pathologic examination revealed mature teratoma.

**Conclusion:**

This is one of the longest long-term reported intervals of a mature teratoma after treatment of a testicular nonseminoma germ cell tumor. This case emphasizes the necessity for follow up of testicular cancer throughout the patient's life.

## Background

The prognosis for nonseminomatous germ cell tumor (NSGCT) of the testis has been dramatically improved by using a treatment protocol of cisplatin-based chemotherapy followed by surgical resection of residual tumor mass. The complete response rate of disseminated germ cell tumor with this protocol is between 70%–80%.

Most patients who relapse do so within the first year of therapy. Late relapse is defined as recurrence after a relapse- free interval of more than two years after completion of primary treatment [1]. The cumulative risk of late relapse in patients appearing relapse -free at two years after first line chemotherapy is 4% in ten years [2].

The incidence of late relapse after cisplatin based chemotherapy of germ cell tumor is related to initial tumor burden and patients with bulky retroperitoneal disease appear to be at an increased risk of late relapse. As tumor markers do not rise in one quarter of late relapses, they should undergo CT scans at annual follow-up evaluations. In the remaining patients, history, physical examination, tumor markers and chest X-ray may allow to detect the majority of late asymptomatic relapses [2].

Herein we report a case of late relapse of a bulky teratocarcinoma, 17 years after completion of treatment as a mature teratoma of the neck and upper thorax.

## Case presentation

**Figure 1:**
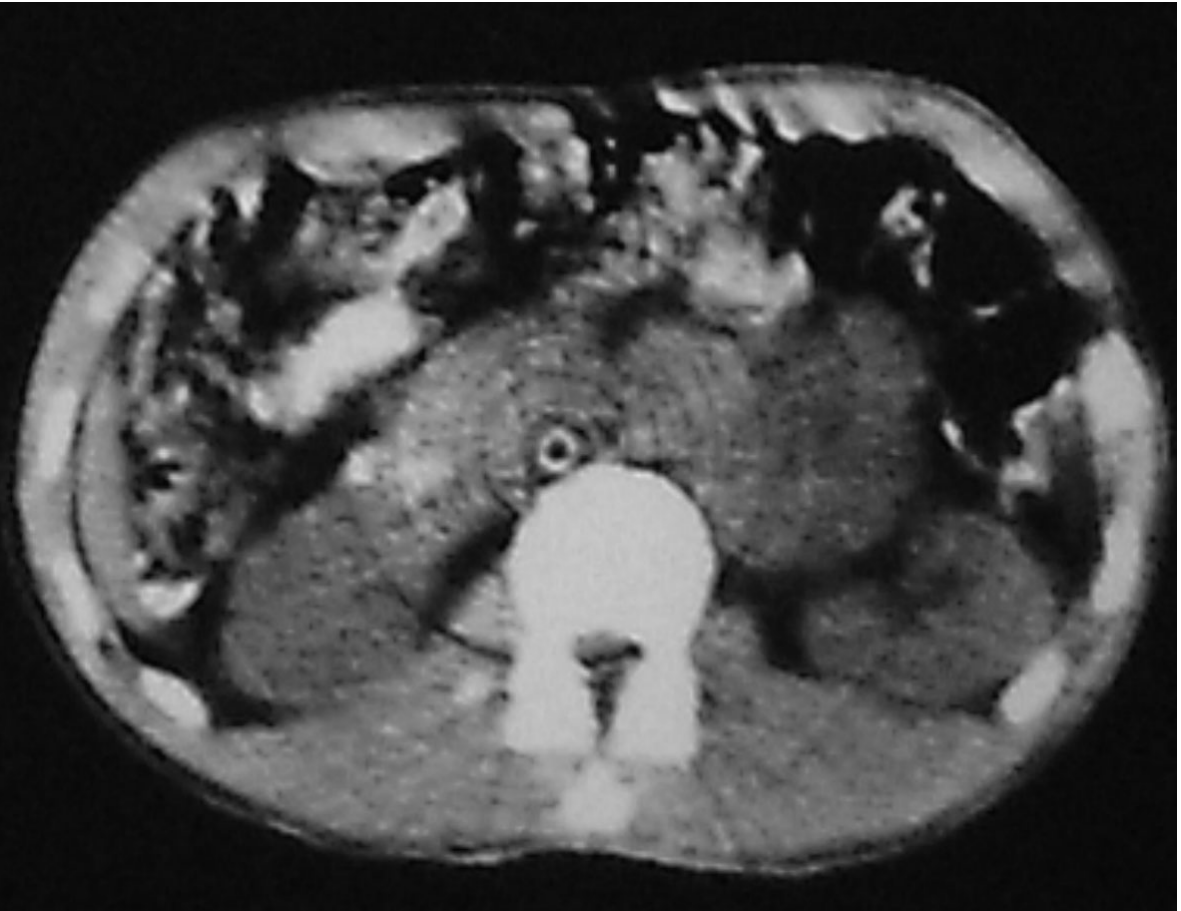
Computed tomography scan showing retroperitoneal residual mass after systemic chemotherapy in 1989.

**Figure 2:**
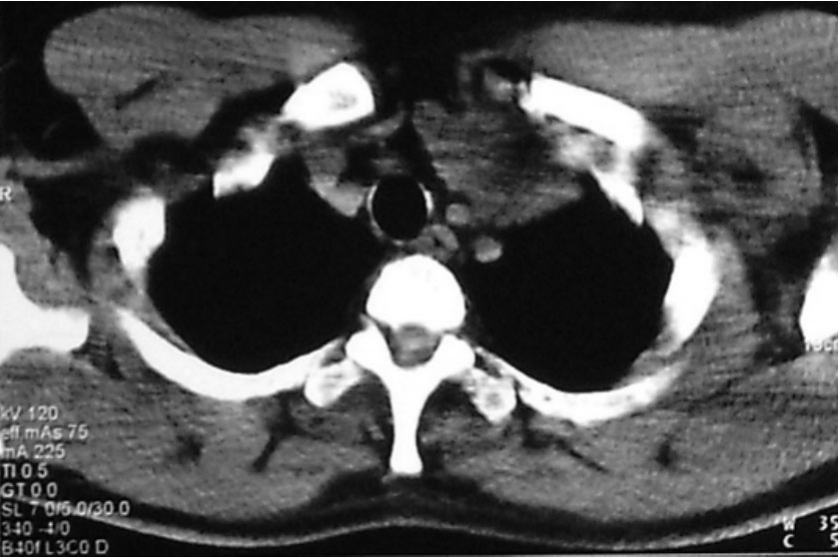
Computed tomography scan showing cervical mass with extension to anterior mediastinum in 2005.

**Figure 3:**
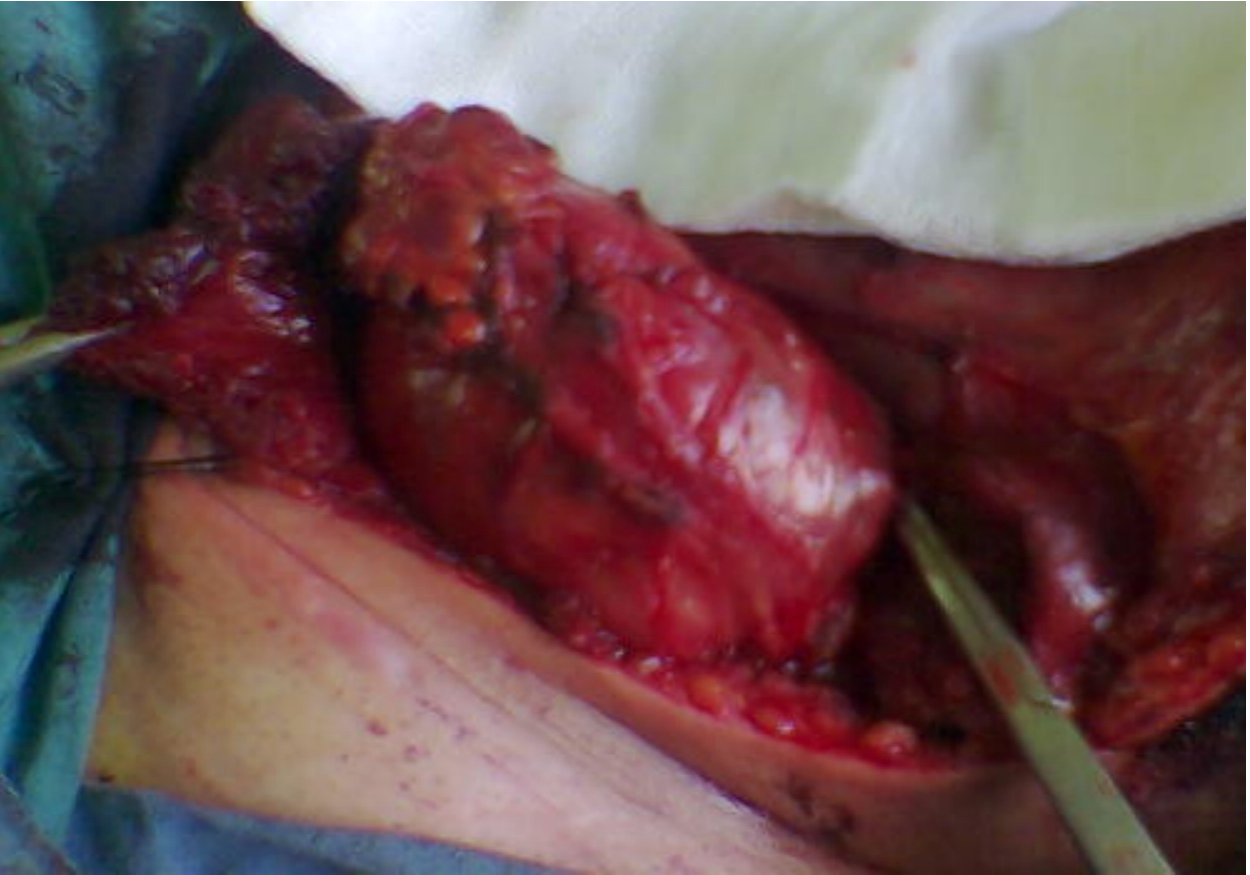
Radical resection of cervical mature teratoma.

A 36 year-old man was admitted to our hospital with chief complaint of left sided neck mass. He had a history of left orchiectomy in 1988 in another center because of testicular teratocarcinoma. At that time he was referred to our center because of huge retroperitoneal mass medial to the left kidney (Figure 1). The first line chemotherapy with bleomycin and etoposide and cisplatin followed by retroperitoneal mass resection revealed mature teratoma and lead to complete remission. He had been in long-term remission for 17 years, but he had regular follow-up for the first 5 years. In November 2005, he was admitted again because of 5 × 5 cm left supraclavicular mass, which had appeared the year before. In physical exam there was no other abnormality and pathologic sign. Serum markers (AFP, beta HCG and LDH) were normal. CT scan of the thorax showed a 5 × 5 × 8 cm lobulated neck mass with extension to anterior mediastinum. (figure 2). The other parts especially paraaortic area were normal in imaging studies. Incisional biopsy of the neck mass confirmed mature teratoma so it was treated by radical surgical resection (figure 3). The specimen consists only of teratoma without any other components especially immature teratoma.

## Discussion

Germ cell tumours, which are in complete remission two years after treatments have a high probability of cure and reports of late relapse, are rare (1.3%–6.2%) [2-6]. Late relapse may occur at any time; of 81 patients treated for late relapse at Indian university, 47 patients (60%) relapsed more than 5 years after the achievement of a complete initial response [7].

The possible mechanisms of development of a late relapse in germ cell tumors include the followings: malignant degeneration of mature teratoma to germinal malignancy, growth of an occult testicular tumor not eliminated by chemotherapy, development of a second primary germinal malignancy, persistent microscopic viable tumor with an atypical less aggressive biologic behaviour [8].

Patient with bulky retroperitoneal disease and patient found to have teratoma following cisplatin-based chemotherapy appear to be at an increased risk of late relapse. Of 51 patients with mature teratoma in resected retroperitoneal residual tumor masses after chemotherapy, 9 patients (17.6%) relapsed. In five patients (9.8 %) relapse resulted from growing mature teratoma [9].

Teratoma is a chemo resistant, nonseminomatous germ cell tumor composed of somatic cell type from two or more germ layers and is derived from a toti potential, malignant precursor cell (embryonal carcinoma or yolk sac tumor). Although teratoma is a benign tumor but its biologic potential is unpredictable and it should be resected completely because it may grow and become unresectable.

Disease free survival following resection of teratoma is related to completeness of resection; therefore, there are significant advantages to surgery with low volume disease. Moreover, there is the risk of malignant transformation of teratoma to carcinoma or sarcoma [10], so unresected teratoma may result in late relapse. A late relapse often shows a slow growth and usually responds to chemotherapy poorly. Complete surgical resection of late relapse is preferred mode of therapy but cure rate are relatively low in patients with viable cancer. In addition, patients with symptomatic disease and patients presenting with visceral metastases carried a poor prognosis [2].

Herein we report a case of late relapse in the left side of neck 17 years after treatment of left testicular teratocarcinoma. The incidence of cervical metastasis in testicular germ cell tumor is about 5% [11]. In germ cell tumour, metastatic disease first involves the retroperitoneal lymph nodes, then the tumor spreads via thoracic duct to the latter's termination near the junction of the left internal jugular and subclavian veins, the area where we resected the mature teratoma of our patient. The mechanisms of late relapse in the neck and upper thorax may include the altered lymphatic drainage from an incompletely resected spermatic cord, a second primary extragonadal tumor focus, or growing of a mature teratoma. Late relapse may occur at any time. In a recent study from Memorial Sloan Kettering Cancer Center the medium time to relapse for 17 patients among 551 patients who had previously complete response to first line chemotherapy was 7.8 years [5]. In a recent analysis of 122 cases of late relapse medium time to relapse was 64.5 months in nonseminomatous germ cell tumours[12]. Lehman et al. reported a case of retroperitoneal mature teratoma 15 years after initial treatment of testicular germ cell tumor [13]. Late relapse was observed up to 32 years after initial treatment [7].

Whereas chemotherapy has only minor curative potential in the treatment of late relapse, patient with localized resectable disease can be cured. Modified neck dissection has a demonstrated valuable role in the treatment of metastatic non-seminomatus germ cell tumours [14].

Our patient has been well with no evidence of disease after resection of cervical mature teratoma until the date of last follow-up (October 2006). However, continued close follow-up of this case is necessary because the large tumor burden of teratoma is a significant adverse factor predictive for further relapse [15]. In our opinion, a reasonable follow up schedule would evaluate the patient monthly in the first year. The frequency of visits should be decreased to 2–3 months in the second and third year, and every 6 months in year 4 and 5, and annually thereafter. Physical examination, tumor markers and radiological examinations are complementary. Annual CT scan can detect late relapse at an asymptomatic phase.

## Conclusion

This case emphasizes the necessity of annual follow-up evaluation of testicular germ cell cancer patient through their life, in order to detect the majority of late relapse at an asymptomatic phase.

## Competing interests

The author(s) declare that they have no competing interest.

## Authors' contributions

**RO **carried out the surgery and drafted the manuscript.

**MA **assisted in operation and participated in drafting the manuscript.

All authors read and approved the final manuscript.

## References

[B1] Terebelo HR, Taylor HG, Brown A, Martin N, Stutz FH, Blom J, Geier L (1983). Late relapse of testicular cancer. J Clin Oncol.

[B2] Gerl A, Clemm C, Schmeller N, Hentrich M, Lamerz R, Willmanns W (1997). Late relapse of germ cell tumors after cisplatin-based chemotherapy. Ann Oncol.

[B3] Oldenburg J, Alfsen GC, Waehre H, Fossa SD (2006). Late recurrences of germ cell malignancies:a population-based experience over three decades. Br J Cancer.

[B4] Borge N, Fossa SD, Ous S, Stenwing AE, Lein HH (1988). Late recurrence of testicular cancer. J Clin Oncol.

[B5] Ronnen EA, Kondagunta GV, Bacik J, Marion S, Bajorin DF, Sheinfeld J, Bosl GJ, Motzer RJ (2005). Incidence of late-relapse germ cell tumor and outcome to salvage chemotherapy. J Clin Oncol.

[B6] Ravi R, Oliver RT, Ong J, Badenoch DF, Fowler CG, Paris AM, Hendry WF (1997). A single-centre observational study of surgery and late malignant events after chemotherapy for germ cell cancer. Br J Urol.

[B7] Baniel J, Foster RS, Gonin R, Messemer JE, Donohue JP, Einhorn LH (1995). Late relapse of testicular cancer. J Clin Oncol.

[B8] De Leo MJ, Greco FA, Hainsworth JD, Johnson DH (1988). Late recurrence in long-term survivors of germ cell neoplasms. Cancer.

[B9] Sonneveld DJ, Sleijfer DT, Koops HS, Keemers-Gels ME, Molennar WM, Hoekstra HJ (1998). Mature teratoma identified after post chemotherapy surgery in patients with disseminated nonseminomatous testicular germ cell tumors:a plea for an aggressive approach. Cancer.

[B10] Motzer RJ, Amsterdam A, Prieto V, Sheinfeld J, Murty vv, Mazumdar M, Bosl GJ, Chaganti RS, Reuter VE (1998). Teratoma with malignant transformation:diverse malignant histologies arising in men with germ cell tumors. J Urol.

[B11] See WA, Laurenzo JF, Dreicer R, Hoffman HT (1996). Incidence and management of testicular carcinoma metastatic to the neck. J Urol.

[B12] Dieckmann KP, Albers P, Classen J, Dewit M, Pichlmeier U, Rick O, Mullerleile U, Kuczyk M (2005). Late relapse of testicular germ cell neoplasms: a descriptive analysis of 122 cases. J Urol.

[B13] Lehmann J, Ritz M, Nurnberg N, Romahn E, Bach S, Kuppers F, Loch T, Stockle M, Weichert-Jacobsen K (2000). Retroperitoneal mature teratoma 15 years after initial treatment of testicular mixed germ cell tumor. Eur Urol.

[B14] Weisberger EC, McBride LC (1999). Modified neck dissection for metastatic nonseminomatous testicular carcinoma. Laryngoscope.

[B15] Loehrer PJ, Mandelbaum I, Hui S, Clark S, Einhorn LH, Williams SD, Donohue JP (1988). Resection of thoracic and abdominal teratoma in patients, after cisplatin-based chemotherapy for germ cell tumor, late results. J Thorac Cardiovas Surg.

